# Reciprocal Relationship Between HDAC2 and P-Glycoprotein/MRP-1 and Their Role in Steroid Resistance in Childhood Nephrotic Syndrome

**DOI:** 10.3389/fphar.2019.00558

**Published:** 2019-05-22

**Authors:** Harshit Singh, Vikas Agarwal, Saurabh Chaturvedi, Durga Prasanna Misra, Akhilesh Kumar Jaiswal, Narayan Prasad

**Affiliations:** ^1^Department of Clinical Immunology, Sanjay Gandhi Post Graduate Institute of Medical Sciences, Lucknow, India; ^2^Department of Nephrology, Sanjay Gandhi Post Graduate Institute of Medical Sciences, Lucknow, India

**Keywords:** P-glycoprotein (P-gp), multidrug resistance-associated protein 1 (MRP-1), histone deacetylase2 (HDAC2), HDAC inhibitor and HDAC stimulator, steroid resistance

## Abstract

**Background:** Reduced HDACs levels have been reported in steroid resistant chronic obstructive pulmonary disease and bronchial asthma patients. P-glycoprotein (P-gp) over expression in peripheral blood mononuclear cells (PBMCs) has been reported in patients with steroid resistant nephrotic syndrome (NS). Whether and how HDACs and P-gp are linked with each other is not clear, especially in NS patients.

**Aim:** To evaluate mRNA expression of P-gp/MRP-1 and HDAC2 in PBMCs of steroid sensitive (SSNS) and steroid resistant nephrotic syndrome (SRNS) patients, and determine the relationship between expression of HDAC2 and P-gp/ MRP-1in NS patients.

**Methods:** Twenty subjects (10 in each group), SSNS (mean age 7.54 ± 3.5 years), and SRNS (mean age 8.43 ± 3.8 years) were recruited. mRNA expression of HDAC2 and P-gp/MRP-1 was studied by quantitative real time PCR. PBMCs were treated with Theophylline, 1 μM, and Trichostatin A, 0.8 μM, for 48 h for induction and suppression of HDAC2, respectively.

**Results:** At baseline, expression of P-gp (4.79 ± 0.10 vs. 2.13 ± 0.12, *p* < 0.0001) and MRP-1 (3.99 ± 0.08 vs. 1.99 ±0.11, *p* < 0.0001) on PBMCs were increased whereas, HDAC2 mRNA levels (2.97 ± 0.15 vs. 6.02 ± 0.13, *p* < 0.0001) were significantly decreased in SRNS as compared to that of SSNS patients. Compared to baseline, theophylline reduced mRNA expression of P-gp and MRP-1 (fold change 2.65 and 2.21, ^*^p < 0.0001 in SRNS) (fold change 1.25, 1.24, ^*^p < 0.0001 in SSNS), respectively. However, it increased the expression of HDAC2 (fold change 5.67, ^*^p < 0.0001 in SRNS) (fold change 6.93, ^*^p < 0.0001 in SSNS). Compared to baseline, TSA treatment increased mRNA levels of P-gp and MRP-1 (fold change 7.51, 7.31, ^*^p < 0.0001 in SRNS) and (fold change 3.49, 3.35, ^*^p < 0.0001 in SSNS), respectively. It significantly decreased the level of HDAC2 (fold change 1.50, ^*^p < 0.0001 in SRNS) (fold change 2.53, ^*^p < 0.0001 in SSNS) patients.

**Conclusion:** Reduced HDAC2 and increased P-gp/MRP-1 activity may play a role in response to steroids in childhood NS. HDAC2 and P-gp/MRP-1 are in reciprocal relationship with each other.

## Introduction

Minimal change disease (MCD) is the most common of all the glomerular diseases in children causing Idiopathic Nephrotic Syndrome (INS). Proteinuria is mainly due to podocyte injury, which may be reversed after steroid therapy (Schnaper, 1989; Lama et al., 2002; Cuzzoni et al., 2015; Colucci et al., 2018). INS is broadly categorized into two groups, steroid-sensitive nephrotic syndrome (SSNS) and steroid-resistant nephrotic syndrome (SRNS) (Noone et al., 2018). Almost half of SSNS patients experience relapses of proteinuria within the first 6 months of stopping steroids. Some of these patients may become steroid resistant later on (Hogg et al., 2000). The treatment of relapsing and SRNS patients is challenging and requires immunosuppressive and potentially toxic drugs (Eddy and Symons, 2003).

Besides changes in histology of kidney from MCD to focal segmental glomerulosclerosis (FSGS), which is relatively less responsive to the steroid, the lack of response to steroid therapy in INS may be limited by inherent or acquired multidrug resistance (MDR) (Prasad et al., 2015). P-glycoprotein (P-gp) and Multi drug resistance associated protein-1(MRP-1) which belongs to ATP-binding cassettes (ABCB1) and (ABCC) sub-families respectively, are energy dependent efflux pump that efflux the steroids out of the cells. This results in to reduced concentration of steroids within the cells, and leads to steroid resistance (Wasilewska et al., 2007; Funaki et al., 2008; Prasad et al., 2015; Badr et al., 2016).

Earlier studies have reported that steroid therapy in INS reduces P-gp expression on PBMCs along with a differential alteration in peripheral blood T-regulatory cells and T-effector cells during remission (Prasad et al., 2015; Colucci et al., 2018).

The anti-inflammatory action of the steroids is mediated by two main mechanisms; transactivation and trans-repression (Barnes, 2010a; Schijvens et al., 2019). During transactivation, steroids induce the transcription of anti-inflammatory genes such as lipocortin, and various mitogen-activated protein kinases (Oakley and Cidlowski, 2013) and others. Whereas during trans-repression, steroids suppresses the transcription of the pro-inflammatory genes by recruiting HDACs to the sites of active transcription of these pro-inflammatory genes (Barnes, 2017). Transcription of any gene is tightly regulated by two class of enzymes present in the nucleus; histone acetyltransferases (HATs) and histone-deacetylases (HDACs). HATs first acetylate the histones of the chromatin structure and open up the tightly coiled DNA structure allowing access to various transcription factors present in the immediate vicinity to bind to their target promoter regions and bring about the transcription of various genes. Once transcription signal is over, HDACs remove the acetyl group from lysine amino acid on histone, allowing the histone to wrap DNA tightly and regulate chromatin structure leading to the modulation of inflammatory gene expression (Urnov and Wolffe, 2001; Thiagalingam et al., 2003).

As steroid suppresses the activation of pro-inflammatory genes by recruiting histone deacetylase 2 (HDAC2) (Ito et al., 2000), a reduction in HDAC2 expression and activity had been linked with increased inflammation in chronic obstructive pulmonary disease (COPD) and bronchial asthma patients (Ito et al., 2002, 2005, 2006). These studies highlight a potential role of HDAC2 in steroid resistance in bronchial asthma and COPD (Kim et al., 2008). However, the role of HDAC2 in steroid resistance in INS is not known. Therefore, this study was undertaken to determine the role of HDAC2 in steroid resistance in INS. Additionally; we explored the relationship between the expression of HDAC2 and P-gp/MRP-1.

## Materials and Methods

### Inclusion/Exclusion Criteria

Children of <2 years and >16 years, and those with a family history of INS were excluded from the study. Definitions of INS, remission, and relapse were based on established criteria according to the International Study for Kidney Diseases in Children (ISKDC). NS in children was defined as proteinuria of 40 mg/m^2^/h or, a ratio of 2 for spot urine protein (milligram)/creatinine (milligram) in the first morning urine sample with hypo-albuminemia (serum albumin <2.5 g/dL) and presence of edema. All children were treated with prednisolone of 60 mg/m^2^ daily for 6 weeks followed by 40 mg/m^2^ alternate day for the next 6 weeks. Samples from patients who achieved remission were taken after 4 weeks of stopping steroid treatment. Remission of NS was defined by urinary protein excretion <4 mg/m^2^/h or urine dipstix nil/trace for three consecutive days. Steroid-resistance was defined as unresponsiveness of 60 mg/m^2^ body surface area per day for 4 weeks of prednisolone therapy. All steroid resistant patients were biopsied and subjected to light microscopy, Immunofluorescence and electron microscopy to confirm MCD. Patients enrolled in this study did not have any (i) underlying secondary causes, they were negative for hepatitis B surface antigen, anti-hepatitis C virus antibody, and human immunodeficiency virus seropositivity. All enrolled subjects had normal serum complement (C3 and C4) levels. Informed consent was obtained from parent or guardian of patients when participants were <7 years and from the participant and parent or guardian of patients when age was 7–18 years as per institute guidelines.

### Human Blood Collection and Peripheral Blood Mononuclear Cells Isolation

In the present study, steroid-sensitive and steroid-resistant subjects (*n* = 10 in both group) were recruited. Demographic and biochemical parameters of the patients are listed in Supplementary Table 1. Peripheral Blood Mononuclear Cells (PBMCs) were isolated from 10 ml venous blood as per the previous report (Sahaf et al., 2008).

### Reagents

Roswell Park Memorial Institute (RPMI) Media (Cat No-R6504), Sodium bicarbonate and Sodium Pyruvate were purchased from Sigma, St Louis, MO, USA. 100X Antibiotic-Antimycotic (Cat No- 15240062) and Fetal Bovine Serum (FBS, Cat No-10270106) was purchased from Gibco-Grand Island, NY, USA. Dimethyl sulfoxide (DMSO, Cat No-D2650) and [3-(4, 5-dimethylthiazol-2-yl)-2, 5-diphenyltetrazolium bromide] (MTT, Cat No-M5655) was purchased from Sigma, St Louis, MO, USA. Theophylline and Trichostatin A (TSA). Histopaque-1077 (Sigma, St. Louis, MO 63103, USA). RNAiso Plus was purchased from Takara Bio Inc., Nojihigashi, Kusatsu, Japan. cDNA Synthesis Kit (cat No-K1632) was purchased from Thermo Fisher Scientific Inc., Bartlesville, OK, USA. LightCycler^®^ 480 SYBR Green I Master was purchased from Roche Diagnostics, Indianapolis, IN, USA.

### Cytotoxicity Assay

The cytotoxicity was performed using the standard 3-(4,5-dimethylthiazol-2-yl)-2,5-diphenyl tetrazolium bromide (MTT) assay. The effect of Theophylline and TSA on the proliferative capacity of PBMCs was quantified using mitochondria-dependent reduction of a tetrazolium dye, MTT to insoluble purple formazan. Briefly, PBMCs were seeded in 96-well plates at 20 × 10^3^ cells per well in RPMI 1640 growth medium with 10% FBS and incubated overnight at 37 °C in a humidified environment containing 5% CO_2_. Culture medium from each well was discarded and replaced with new media on the third day. On the fourth day, cells were synchronized with a low serum medium for 24 h. Following synchronization, cells were incubated with TSA for 24 h. Following incubation at 37°C in 5% CO_2_ for 24 h, the medium was discarded and MTT solution was added to each well (final concentration, 500 μg/ml) after 3 h of incubation at 37°C. The supernatant was then removed and 150 μl of fresh DMSO was added to each well. The absorbance of the solution was read at 570 nm with a microplate reader (Bio-Rad 550; Bio-Rad, Japan) (Xu et al., 2012).

### Cell Culture Conditions and Stimulation of Cells

To test the expression levels of P-gp and MRP-1, PBMCs were isolated and cultured for 24 h. PBMCs were maintained at 2.5 × 10^6^ cells/ml concentration in RPMI 1640 medium with 10% FBS and 1% Penicillin/ Streptomycin/Amphotericin-B at 37°C in a humidified atmosphere with 5% CO_2_ for a period of 24 h for stable growth (Sahaf et al., 2008). Afterward, cells were treated with of 1 μM of Theophylline and 0.8 μM of TSA for a period of 48 h. After 48 h of incubation, the cultures were transferred to 1.5 ml centrifuge tubes and centrifuged to pellet cells down. Cell pellets were used as a source for RNA extraction. mRNA levels of P-gp/MRP-1 were quantified by real-time PCR, normalized to housekeeping Glyceraldehyde 3-phosphate dehydrogenase (GAPDH).

### Real Time Quantitative PCR Analysis

RNA was extracted from PBMCs incubated with HDAC2 stimulator (Theophylline) and inhibitor (Trichostatin A) for 48 h using RNAiso Plus (Trizol method), and a total of 1 μg of RNA was processed for cDNA synthesis using cDNA Synthesis Kit (Thermo Fisher Scientific Inc., Bartlesville, OK, USA) as per the manufacturer's protocol. We performed real-time PCR reactions for each cDNA sample in triplicate using LightCycler^®^ 480 SYBR Green I Master and gene-specific primer pairs for P-gp, MRP-1, HDAC2, and GAPDH (listed in Supplementary Table 2). The PCR cycling was as follows: 50°C for 2 min for 1 cycle, 95°C for 10 min for 1 cycle, 95°C for 15 s, 60°C for 1 min for 40 cycles in light cycler LC480 (Roche, USA). We expressed semi-quantitative real-time PCR data for each target gene as 2–ΔΔCt Relative Quantitation (RQ) vs. endogenous GAPDH, with error bars representing the standard error of the mean for triplicate reactions and the data being expressed as fold change (Chaturvedi et al., 2018).

### Statistical Analysis

Each experiment was performed in triplicates and an average of all experiments were represented as mean±SEM. Differences in the various parameters between groups were evaluated by students *t*-test. Results were considered significant if the *P-*value was ≤ 0.05. The experiment was performed in three independent series.

## Results

### Expression of P-Gp, MRP-1, and HDAC2 in SRNS and SSNS Patients

**Figure 1:**
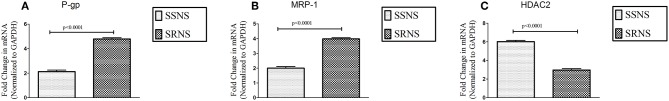
Expression of P-gp, MRP-1 and HDAC2 on unstimulated PBMCs of SRNS and SSNS patients. Peripheral Blood Mononuclear Cells were isolated from SRNS and SSNS patients and the mRNA levels of P-gp, MRP-1 and HDAC2 were quantified by real-time PCR technique **(A–C)**. The experiments are representative of three independent series. Pooled data of all the experiments are represented as mean ± SEM. Significant differences compared to control were indicated by *p* < 0.05.

Expression of P-gp (4.79 ± 0.10 vs. 2.13 ± 0.12, *p* < 0.0001) and MRP-1 (3.99 ± 0.08 vs. 1.99 ± 0.11, *p* < 0.0001) on PBMCs was increased in SRNS as compared to SSNS. HDAC2 mRNA levels were significantly decreased in SRNS as compared to SSNS patients (2.97 ± 0.15 vs. 6.02 ± 0.13, *p* < 0.0001). Thus, we observed that mRNA levels of HDAC2 were reduced while, P-gp/MRP-1 levels were increased in PBMCs of SRNS patients (Figures 1A–C). Compared to baseline levels, mRNA expression of P-gp/MRP-1 in three SSNS patients was significantly increased whereas that of HDAC2 was significantly decreased at the time of relapse (Supplementary Figures 1A–C). Similarly, compared to baseline, mRNA expression of P-gp/MRP-1 was significantly reduced and that of HDAC2 was significantly increased in four SRNS patients who achieved remission after treatment with Tacrolimus therapy (Supplementary Figures 2A–C).

### Optimization of Doses of Theophylline and Trichostatin a in PBMCs of SRNS and SSNS Patients

Theophylline has been reported to induce a 6-fold increase in HDAC activity in macrophages of COPD patients [21]. PBMCs of SRNS and SSNS were incubated with Theophylline and TSA respectively, at various biologically relevant concentrations [17, 21] (Supplementary Figures 3A,B, 4A,B, respectively). Percentage viability for Theophylline at 1 μM and TSA at 0.8 μM was 80%. Higher concentrations of both resulted in decreased viability. Thus, a concentration of 1 μM for Theophylline and 0.8 μM for TSA were used in further experiments.

### Effect of HDAC Stimulator on Multidrug Resistance Proteins in PBMCs of SRNS and SSNS Patients

**Figure 2:**
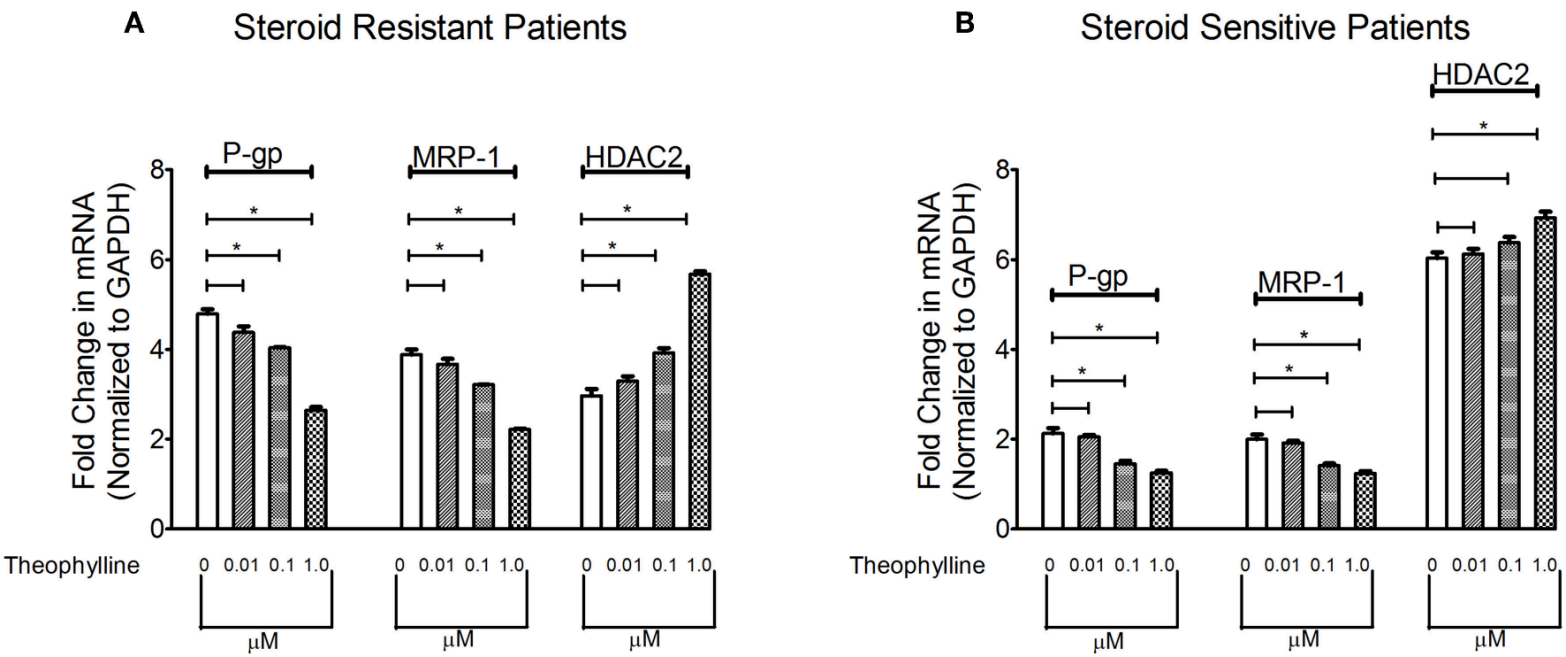
Effect of HDAC2 stimulator Theophylline on expression levels of P-gp, MRP-1 and HDAC2 in SRNS and SSNS patients **(A,B)**. Quantitative real-time PCR of P-gp, MRP-1 **(A)** and HDAC2 **(B)** in peripheral blood mononuclear cells of SRNS and SSNS patients treated with different concentrations of Theophylline (0, 0.01, 0.1, 1.0 μM) for 48 h. The experiments are representative of three independent series. Pooled data of all the experiments are represented as mean±SEM. Significant differences compared to control are indicated by *p < 0.05.

To determine the effect of HDAC stimulator on P-gp/MRP-1, PBMCs from patients with SRNS and SSNS were cultured with theophylline. Compared to baseline, theophylline decreased mRNA levels of P-gp/MRP-1 in PBMCs of SRNS and SSNS patients with maximal induction at 1 μM (Figure 2). Additionally, HDAC2 mRNA expression increased maximally at 1 μM concentration of theophylline as compared to the baseline (Figure 2). Thus, our data indicate that activation of HDAC2 suppressed expression of P-gp/MRP-1 in PBMCs of SRNS and SSNS patients.

### The Effect of HDAC Inhibitor at Different Concentrations on Multidrug Resistance Proteins in PBMCs of SRNS and SSNS Patients

**Figure 3:**
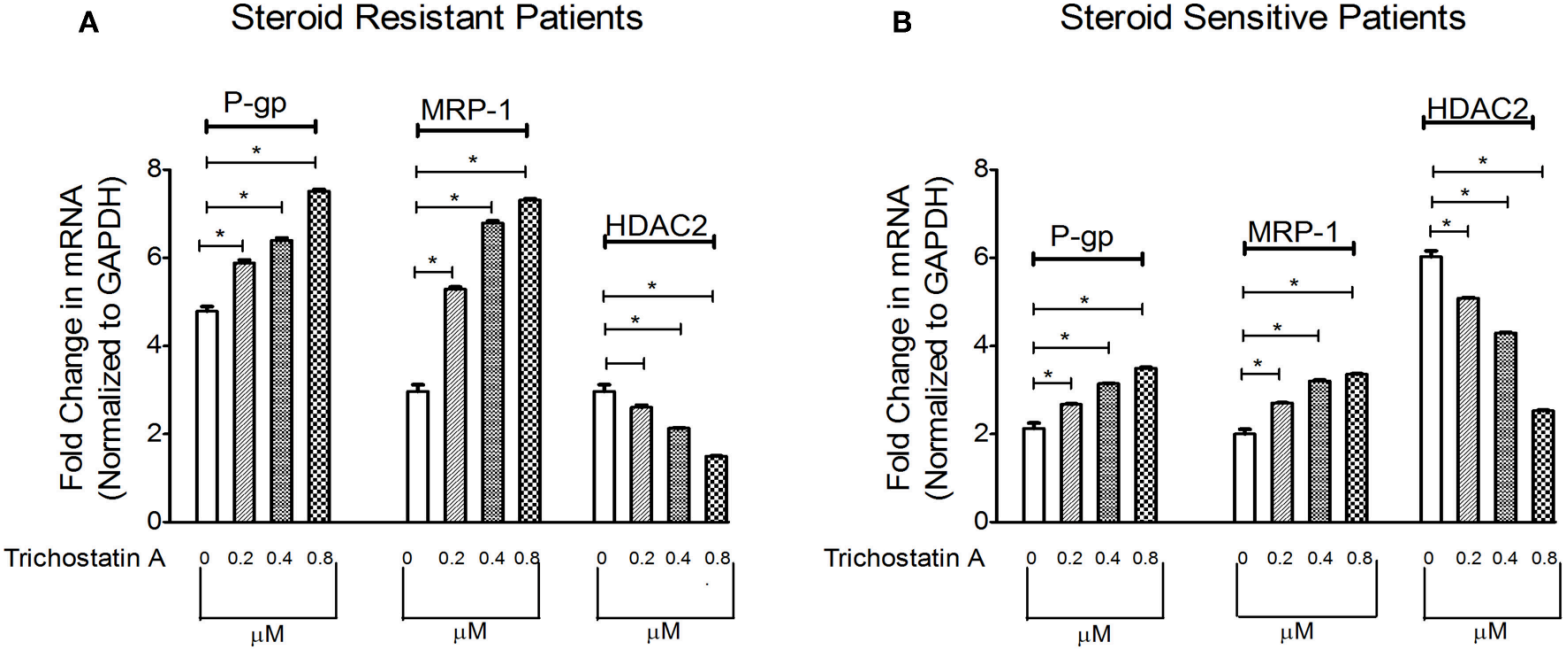
Effect of HDAC2 inhibitor Trichostatin A expression levels of P-gp, MRP-1 and HDAC2 in SRNS and SSNS patients **(A,B)**. Quantitative real-time PCR of P-gp, MRP-1, and HDAC2 in peripheral blood mononuclear cells of SRNS and SSNS patients treated with different concentrations of Trichostatin A (0, 0.2, 0.4, 0.8 μM) for 48 h. The experiments are representative of three independent series. Pooled data of all the experiments are represented as mean ± SEM. Significant differences compared to control were indicated by *p < 0.05.

Next, we determined whether inhibition of HDAC can modulate levels of P-gp/MRP-1. Compared to baseline, TSA increased mRNA levels of P-gp/MRP-1 in PBMCs of SRNS and SSNS patients with maximal induction at 0.8 μM. (Figure 3). Additionally, HDAC2 mRNA expression decreased maximally at 0.8 μM (Figure 3) compared to the baseline. Thus, our data indicate that inhibition of HDAC2 leads to increased expression of P-gp/MRP-1 in PBMCs of SRNS and SSNS patients.

## Discussion

There was increased expression of P-gp/MRP-1 and reduced expression of HDAC2 in PBMCs of patients with SRNS as compared to SSNS. Expression of P-gp/MRP-1 and levels of mRNA of HDAC2 reflected the state of remission or disease activity in INS children. Inducers of HDAC2 had a downregulating effect on P-gp/MRP-1, whereas, inhibitors of HDAC2 up-regulated the expression of P-gp/MRP-1, suggesting a reciprocal relationship between the two.

In INS, an imbalance between pro-inflammatory mediators such as Interferon γ, Interleukin-2, Interleukin- 4, Interleukin-17 (IFNγ, IL2, IL4, IL17) and anti-inflammatory transforming growth factor beta 1, Interleukin 10 (TGF-β1, IL-10) cytokines, respectively have been reported to be responsible for inflammatory condition leading to disease phenotype (Lama et al., 2002; Prasad et al., 2015). Majority of the pro-inflammatory cytokines are secreted by T helper cell 1 and T helper cell 17 (Th1, Th17 cells) (Schnaper, 1989; Lama et al., 2002), whereas, anti-inflammatory cytokines are secreted by Th2 and regulatory T cells (T-regs). These cytokines genes are regulated by HDAC2. Reduced HDAC2 levels in COPD and Bronchial Asthma had been linked with increased expression of pro-inflammatory cytokines genes via nuclear factor kappa-light-chain-enhancer of activated B cells (NFκB) mediated pathways (Barnes, 2009). Thus, reduced HDAC2 levels may contribute to an increase in pro-inflammatory cytokines milieu, leading to steroid resistance in INS patients.

Increased P-gp expression, activity as well as single nucleotide polymorphism (G2677T/A) have been reported to mediate steroid resistance in INS and other autoimmune diseases (Jafar et al., 2011; Prasad et al., 2014). P-gp/MRP-1 over expression and increased functionality reduce the bioavailability of steroids to the cells. In the present study, increased P-gp/MRP-1 and reduced HDAC2 expressions were observed in SRNS patients, suggesting a possible role for both in mediating steroid resistance. Selective knockdown of HDAC1, HDAC2, and a combination of both HDAC1 plus HDAC2 has been reported to increase the expressions of P-gp, MRP-1, and MRP2 in cell lines derived from human colorectal adenocarcinomas (HCT-8 and HCT-116). Additionally, overexpression of HDAC1 and HDAC2 significantly reduced the expression of P-gp, MRP-1, and MRP2 leading to a reversal of multidrug resistance (Xu et al., 2012). The results of the present study are in accordance with those of Xu et al. however the significance of the present study lies in the fact that is was carried out on PBMCs of INS patients as against cancer cell lines in the former study.

Similar to our study, there are reports that demonstrate that HDAC inhibitors Suberoyl anilide hydroxamic acid (SAHA) and TSA increased P-gp protein expression and its encoding gene ABCB1 mRNA expression (Wang et al., 2016). The molecular mechanisms involved in HDAC inhibitors induced up-regulation of P-gp expression is still unknown. However, it has been proposed that apicidin mediated induction of P-gp expression requires mechanism other than DNA methylation regulation (Kim et al., 2009). Similarly, another study utilizing placental cells showed that HDAC2 was involved in transcriptional repression of P-gp, and dissociation of HDAC2 from the promoter region may lead to recruitment of P300, P300/CBP-associated factor (PCAF) and nuclear transcription factor Y (NF-Y) to the promoter region via acetylation of SP1 which may result in induction of P-gp (Duan et al., 2017).

**Figure 4:**
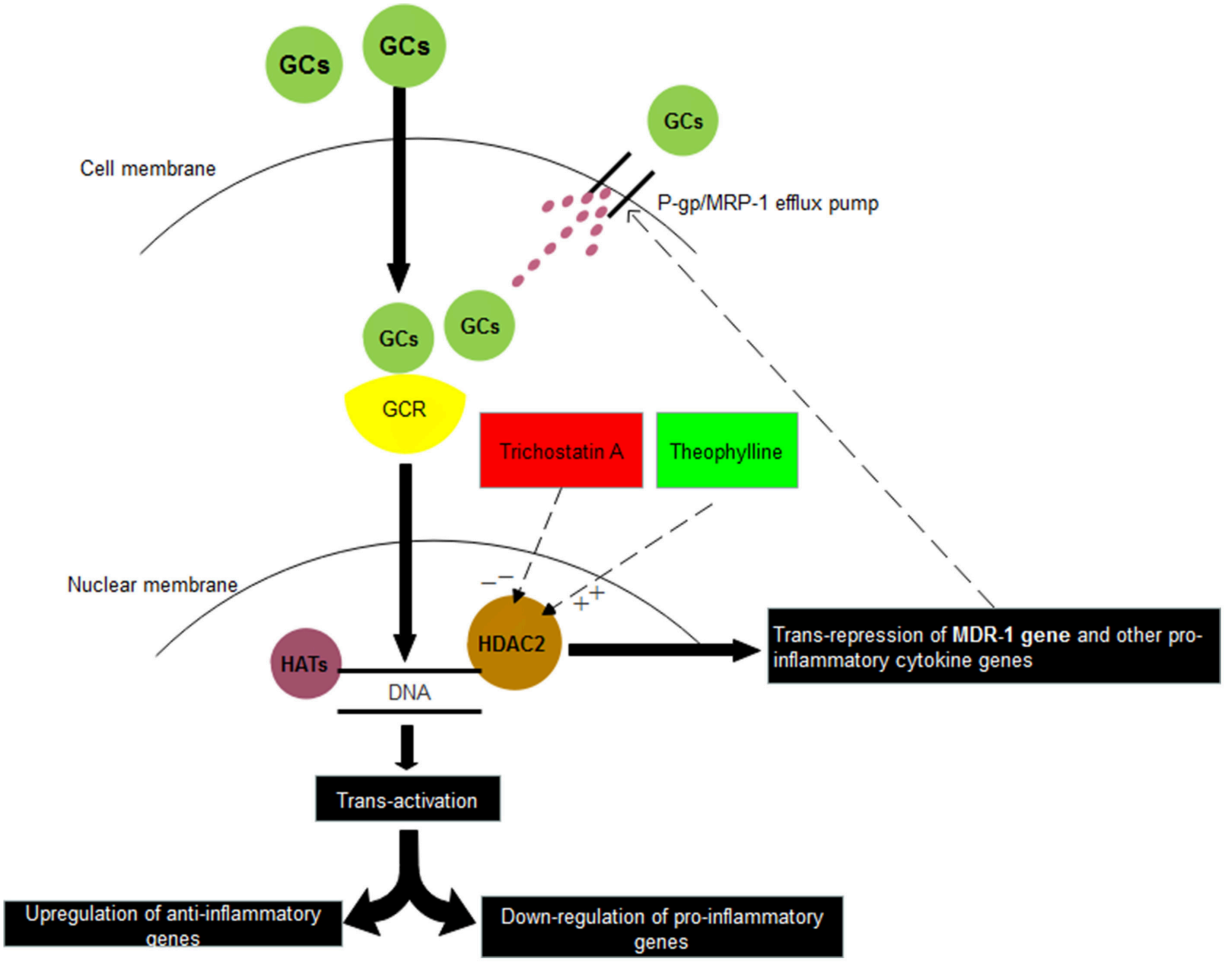
Relationship between corticosteroids and HDAC2. Glucocorticoids (GCs) on entering into the cell binds with Glucocorticoid receptor (GCR) present in the cytosol. Thereafter, GC-GCR complex enters inside the nucleus and binds to Glucocorticoid Receptor Elements (GREs) present on the promotor regions of various genes. Depending upon its binding to either positive GREs or negative GREs, transactivation or trans-repression of genes is resulted. In another *trans-*repression mechanism, GC-GCR complex induces the recruitment of HDAC2, which binds to GREs of certain genes and results in deacetylation of MDR-1 gene, other pro-inflammatory genes. Thus, inhibitors of HDAC2 (Trichostatin A) may suppress the steroid response whereas stimulators of HDAC2 (Theophylline) may increase the steroid response. HAT: Histone Acetylases, HDAC2: Histone Deacetylases 2.

Theophylline has been reported to reverse glucocorticoid resistance via the activation of HDAC2 in COPD macrophages (Cosio et al., 2004; Barnes, 2010b). In INS similar to the other diseases we observed that HDAC2 mRNA expression in PBMCs was reduced markedly in SRNS as compared to SSNS patients. However, treatment with theophylline restored the expression of HDAC2 and down-regulated the expression of P-gp/MRP-1. Presently treatment of SRNS is by use of immunosuppressive and potentially toxic drugs like calcineurin inhibitors, azathioprine, and mycophenolate mofetil. In comparison to these toxic drugs, theophylline or other inducers of HDAC2 seem safe, especially during childhood. Thus, inducers of HDAC2 may have the potential to contribute to the therapeutics of the SRNS patients (Figure 4).

## Conclusion

Reduced HDAC2 and increased P-gp/MRP-1 expression may play a role in steroid response in childhood NS. HDAC2 and P-gp/MRP-1 are in reciprocal relationship with each other. Inducer of HDAC2 may have potential role in the therapeutics of SRNS patients.

## Ethics Statement

This study was carried out in accordance with the recommendations of Institutional Ethics Committee, Sanjay Gandhi Post Graduate Institute of Medical with written informed consent from all subjects. All subjects gave written informed consent in accordance with the Declaration of Helsinki. The protocol was approved by the Institutional Ethics Committee, Sanjay Gandhi Post Graduate Institute of Medical; Sciences (Ethics Cell No: 2016/76/IMP/EXP).

## Author Contributions

HS,VA, SC, and NP contributed to the conception and design, acquisition of data, analysis and interpretation of the data and final approval of the version to be published. HS, VA, SC and DM drafted the article. NP, AJ, and VA critically revised the article for important intellectual content. HS, VA, SC, and NP agree to be accountable for all aspects of the work in ensuring that questions related to the accuracy or integrity of any part of the work are appropriately investigated and resolved.

### Conflict of Interest Statement

The authors declare that the research was conducted in the absence of any commercial or financial relationships that could be construed as a potential conflict of interest.

## Supplementary Material

Supplementary Figure 1 Expression of P-gp, MRP-1 and HDAC2 on unstimulated PBMCs of SSNS patients (*n* = 3) during remission and their subsequent relapse. Peripheral Blood Mononuclear Cells were isolated from SSNS patients (*n* = 3) during remission and their subsequent relapse. mRNA levels of P-gp, MRP-1 and HDAC2 were quantified by real-time PCR technique **(A–C)**. The experiments are representative of three independent series. Pooled data of all the experiments are represented as mean ± SEM. Significant differences were indicated by *p* < 0.05.

Supplementary Figure 2 Expression of P-gp, MRP-1 and HDAC2 on unstimulated PBMCs of SRNS patients (*n* = 4) during active disease and their subsequent remission. Peripheral Blood Mononuclear Cells were isolated from SRNS patients (*n* = 4) during active disease and their subsequent remission. mRNA levels of P-gp, MRP-1 and HDAC2 were quantified by real-time PCR technique **(A–C)**. The experiments are representative of three independent series. Pooled data of all the experiments are represented as mean ± SEM. Significant differences were indicated by *p* < 0.05.

Supplementary Figure 3 Effect of Theophylline and on viability of PBMCs isolated from SRNS and SSSNS patients. **(A,B)**. Peripheral Blood Mononuclear Cells were incubated with increasing doses of HDAC2 stimulator and HDAC2 inhibitor. The experiments are representative of three independent series. Pooled data of all the experiments are represented as mean ± SEM. Significant differences compared to control were indicated by ^*^*p* < 0.05; ^**^*p* < 0.01.

Supplementary Figure 4 Effect of Trichostatin A on viability of PBMCs isolated from SRNS and SSNS patients **(A,B)**. Peripheral Blood Mononuclear Cells were incubated with increasing doses of HDAC2 stimulator and HDAC2 inhibitor. The experiments are representative of three independent series. Pooled data of all the experiments are represented as mean ± SEM. Significant differences compared to control were indicated by ^*^*p* < 0.05; ^**^*p* < 0.01.

Supplementary Table 1 Demographic and biochemical parameters of the patients with nephrotic syndrome.

Supplementary Table 2 Primer sequences for P-glycoprotein, Multidrug resistance-associated protein 1 (MRP-1), Histone Deacetylase2, Glyceraldehyde 3-phosphate dehydrogenase.
