# Supplementary figures and images for: Reciprocal Relationship Between HDAC2 and P-Glycoprotein/MRP-1 and Their Role in Steroid Resistance in Childhood Nephrotic Syndrome
Source: Front Pharmacol. 2019 May 22;10:558. doi: 10.3389/fphar.2019.00558 (PMC6540828; doi:10.3389/fphar.2019.00558)

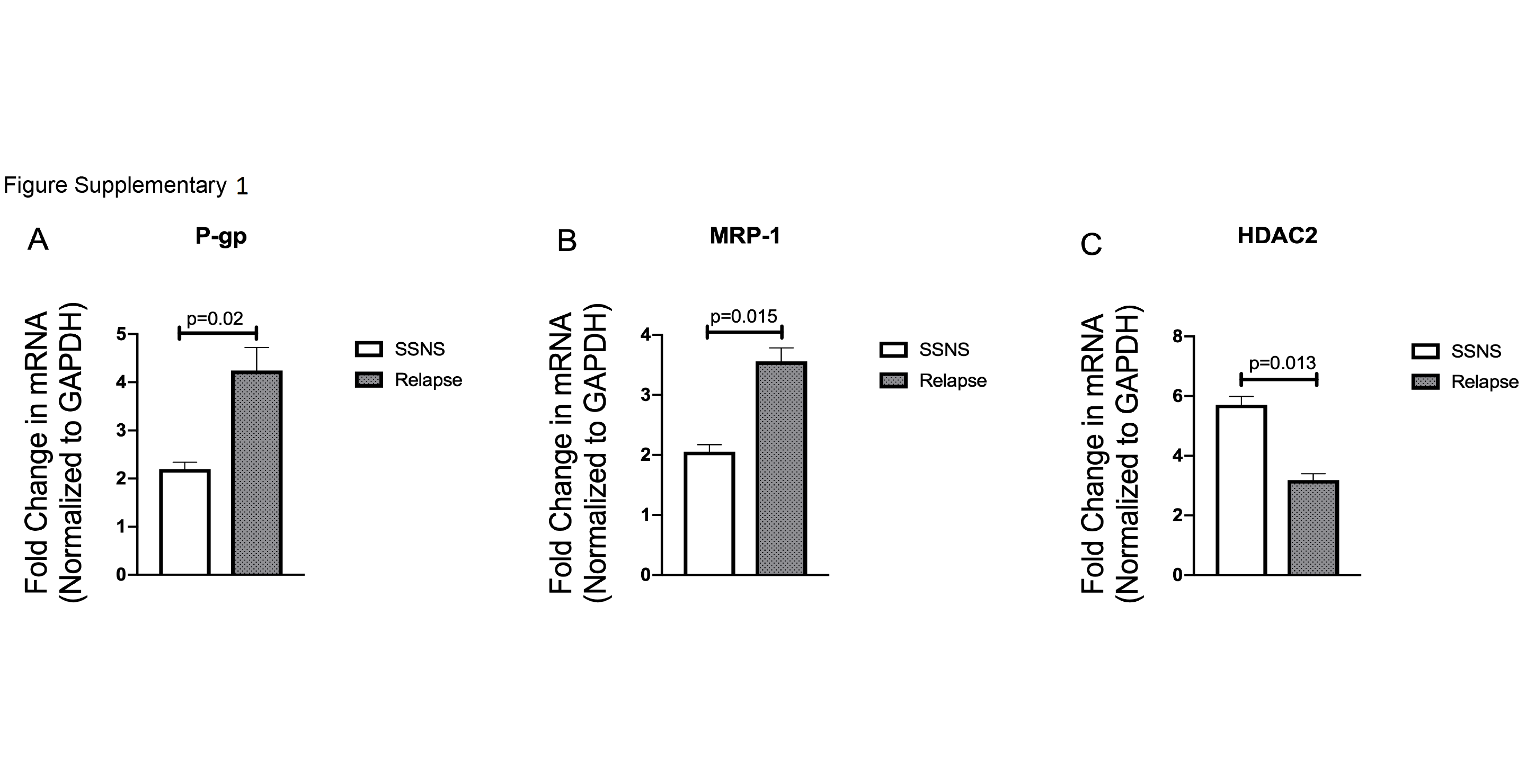

Supplement: Supplementary Figure 1 — Expression of P-gp, MRP-1 and HDAC2 on unstimulated PBMCs of SSNS patients (n = 3) during remission and their subsequent relapse. Peripheral Blood Mononuclear Cells were isolated from SSNS patients (n = 3) during remission and their subsequent relapse. mRNA levels of P-gp, MRP-1 and HDAC2 were quantified by real-time PCR technique (A–C). The experiments are representative of three independent series. Pooled data of all the experiments are represented as mean ± SEM. Significant differences were indicated by p < 0.05. [file Image_1.tif]

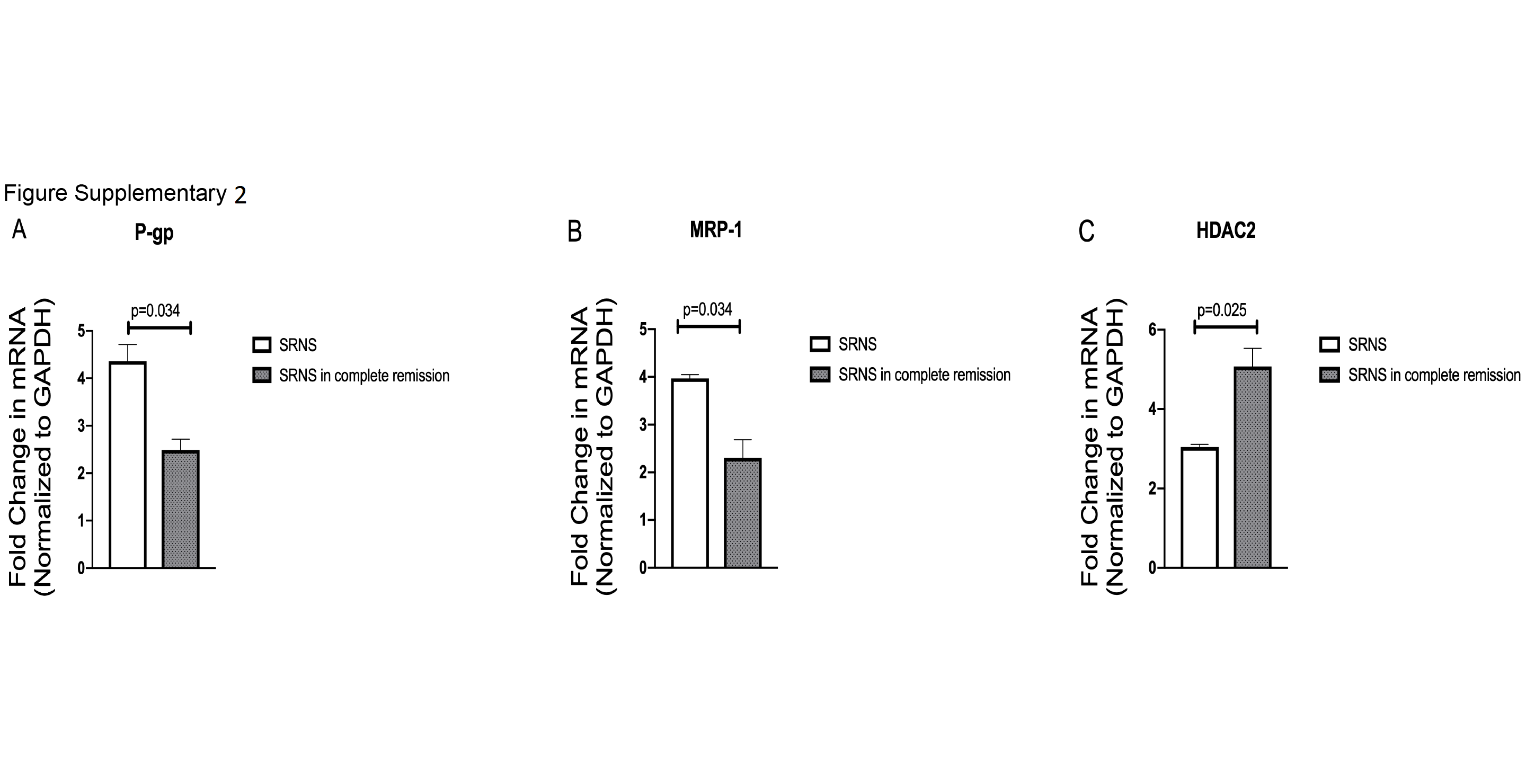

Supplement: Supplementary Figure 2 — Expression of P-gp, MRP-1 and HDAC2 on unstimulated PBMCs of SRNS patients (n = 4) during active disease and their subsequent remission. Peripheral Blood Mononuclear Cells were isolated from SRNS patients (n = 4) during active disease and their subsequent remission. mRNA levels of P-gp, MRP-1 and HDAC2 were quantified by real-time PCR technique (A–C). The experiments are representative of three independent series. Pooled data of all the experiments are represented as mean ± SEM. Significant differences were indicated by p < 0.05. [file Image_2.tif]

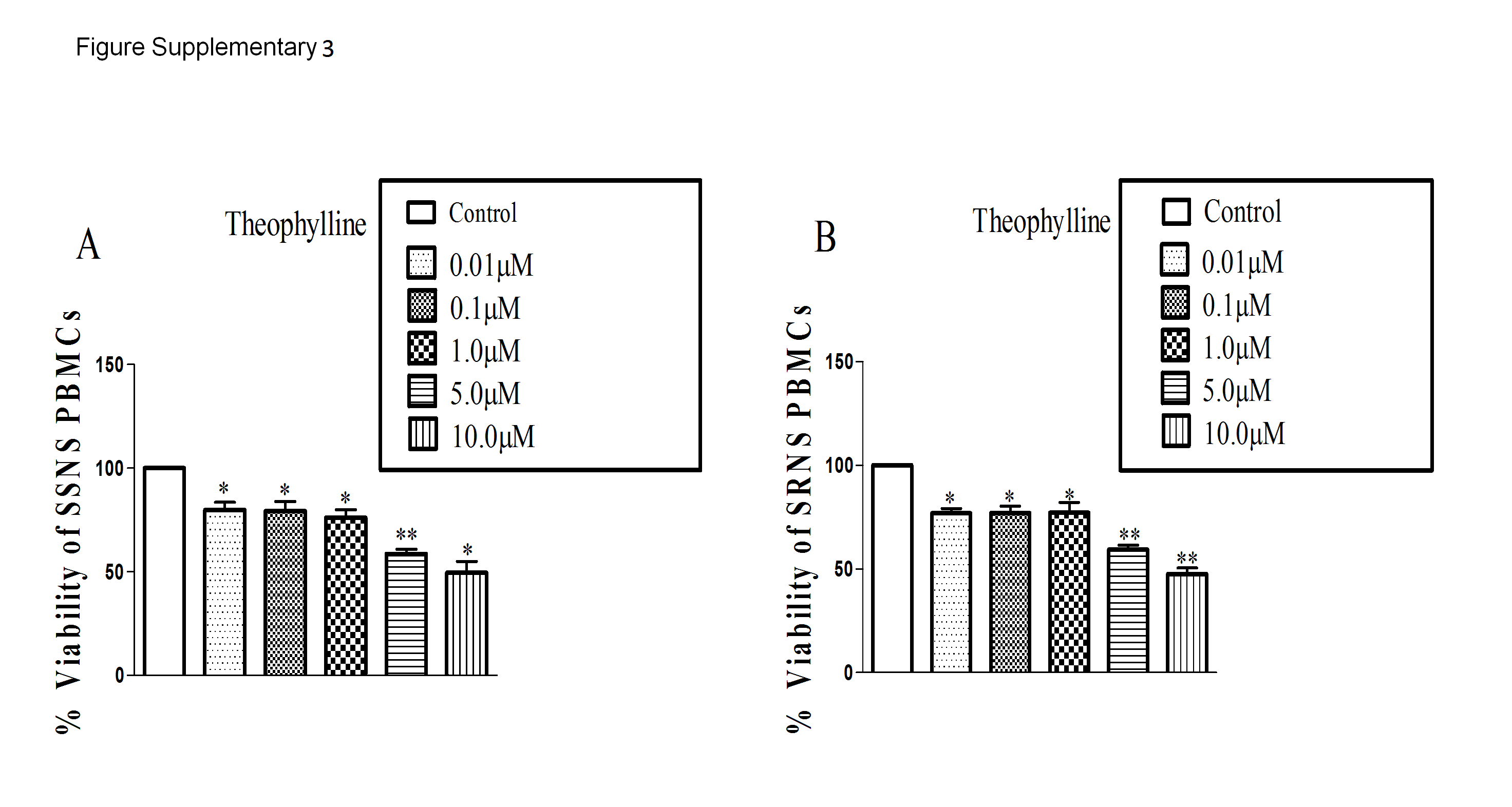

Supplement: Supplementary Figure 3 — Effect of Theophylline and on viability of PBMCs isolated from SRNS and SSSNS patients. (A,B). Peripheral Blood Mononuclear Cells were incubated with increasing doses of HDAC2 stimulator and HDAC2 inhibitor. The experiments are representative of three independent series. Pooled data of all the experiments are represented as mean ± SEM. Significant differences compared to control were indicated by *p < 0.05; **p < 0.01. [file Image_3.tif]

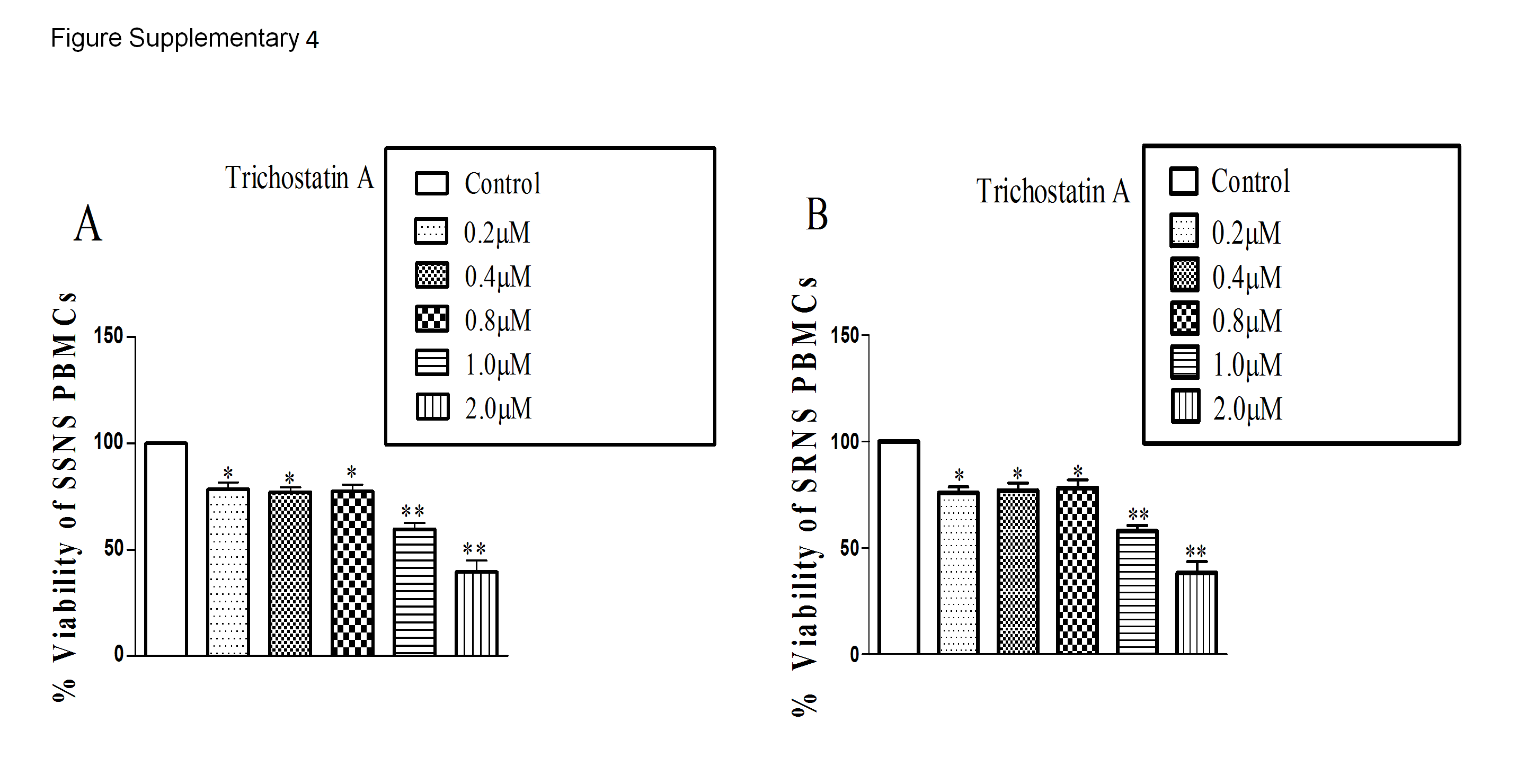

Supplement: Supplementary Figure 4 — Effect of Trichostatin A on viability of PBMCs isolated from SRNS and SSNS patients (A,B). Peripheral Blood Mononuclear Cells were incubated with increasing doses of HDAC2 stimulator and HDAC2 inhibitor. The experiments are representative of three independent series. Pooled data of all the experiments are represented as mean ± SEM. Significant differences compared to control were indicated by *p < 0.05; **p < 0.01. [file Image_4.tif]
