# Supplementary material for: Reciprocal Relationship Between HDAC2 and P-Glycoprotein/MRP-1 and Their Role in Steroid Resistance in Childhood Nephrotic Syndrome
Source: Front Pharmacol. 2019 May 22;10:558. doi: 10.3389/fphar.2019.00558 (PMC6540828; doi:10.3389/fphar.2019.00558)
Supplement: Supplementary Table 1 — Demographic and biochemical parameters of the patients with nephrotic syndrome. [file Table_1.DOCX]

**SUPPLEMENTARY TABLE 1. Demographic and Clinical Details of Patients with nephrotic syndrome**

| **Demographic Profile** | **Remission (n=10)** | **Resistant (n=10)** | **p value** |
| --- | --- | --- | --- |
| **Age (Years)** | 7.60±4.22 | 9.82±3.71 | 0.215 |
| **Height (cm)** | 114.6±16.9 | 120.00±21.21 | 0.530 |
| **Weight (kg)** | 21.80±5.49 | 25.45±4.94 | 0.125 |
| **Age of onset of disease (Years)** | 8.77±3.74 | 4.20±2.52 | 0.004 |
| **Biochemical Parameters** | | | |
| **Haemoglobin (mg/dl)** | 12.55±1.1 | 12.33±1.2 | 0.690 |
| **Systolic Blood Pressure (mmHg)** | 95.00±5.35 | 96.45±7.81 | 0.628 |
| **Diastolic Blood Pressure (mmHg)** | 70.40±9.41 | 71.18±8.79 | 0.846 |
| **24hrs Urinary Protein/ Creatinine ratio (g/24hous)** | 0.13±0.06 | 3.47±0.90 | <0.001 |
| **Serum Blood Urea Nitrogen (mg/dl)** | 12.14±5.80 | 11.35±8.25 | 0.805 |
| **Serum Creatinine (mg/dl)** | 0.86±0.22 | 0.99±0.28 | 0.235 |
| **Total Cholesterol (TC) (mg/dl)** | 115.00±61.21 | 412.00±211.90 | <0.001 |
| **Triglyceride (TG) (mg/dl)** | 130.20±65.22 | 429.54±131.88 | <0.001 |
