# Supplementary material for: Reciprocal Relationship Between HDAC2 and P-Glycoprotein/MRP-1 and Their Role in Steroid Resistance in Childhood Nephrotic Syndrome
Source: Front Pharmacol. 2019 May 22;10:558. doi: 10.3389/fphar.2019.00558 (PMC6540828; doi:10.3389/fphar.2019.00558)
Supplement: Supplementary Table 2 — Primer sequences for P-glycoprotein, Multidrug resistance-associated protein 1 (MRP-1), Histone Deacetylase2, Glyceraldehyde 3-phosphate dehydrogenase. [file Table_2.DOCX]

**SUPPLEMENTARY TABLE 2. Quantitative Polymerase Chain reaction primer sequences**

|  |
| --- |

**Target Gene (Human) Sequence (5’-3’)**

|  |
| --- |

HDAC2 F: TGACAAACCAGAACACTCCAG

R: CTTCTCCATCTTCATCTCCACTG

P-gp F: AGGAAGCCAATGCCTATGACTTTA

R: CAACTGGGCCCCTCTCTCTC

MRP1 F: CTTGGCCACGTACATTAACATGAT

R: CCGATTGTCTTTGCTCTTCATG

GAPDH F: CAAGGTCATCCACGACCACT

R: CCAGTGAGTTTCCCGTTCAG

|  |
| --- |
